# Supplementary figures and images for: Relationships between puroindoline A-prolamin interactions and wheat grain hardness
Source: PLoS One. 2020 Sep 29;15(9):e0225293. doi: 10.1371/journal.pone.0225293 (PMC7523994; doi:10.1371/journal.pone.0225293)

**S1 Figure.**

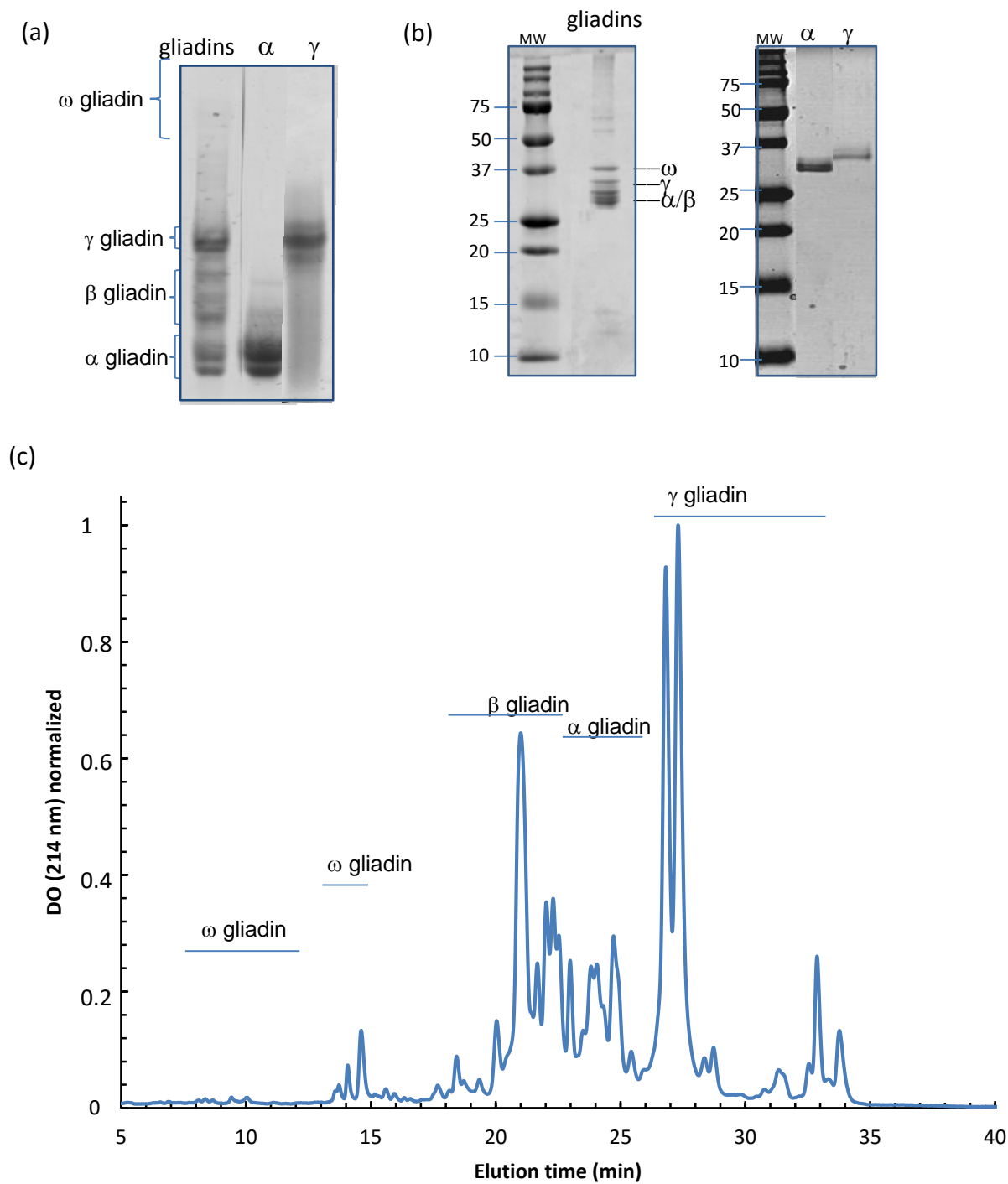

Supplement: S1 Fig — (a) acid-PAGE (b) SDS-PAGE, and (c) C18-PFP RP-HPLC. (PDF) [file pone.0225293.s001.pdf]

**S2 Figure.**

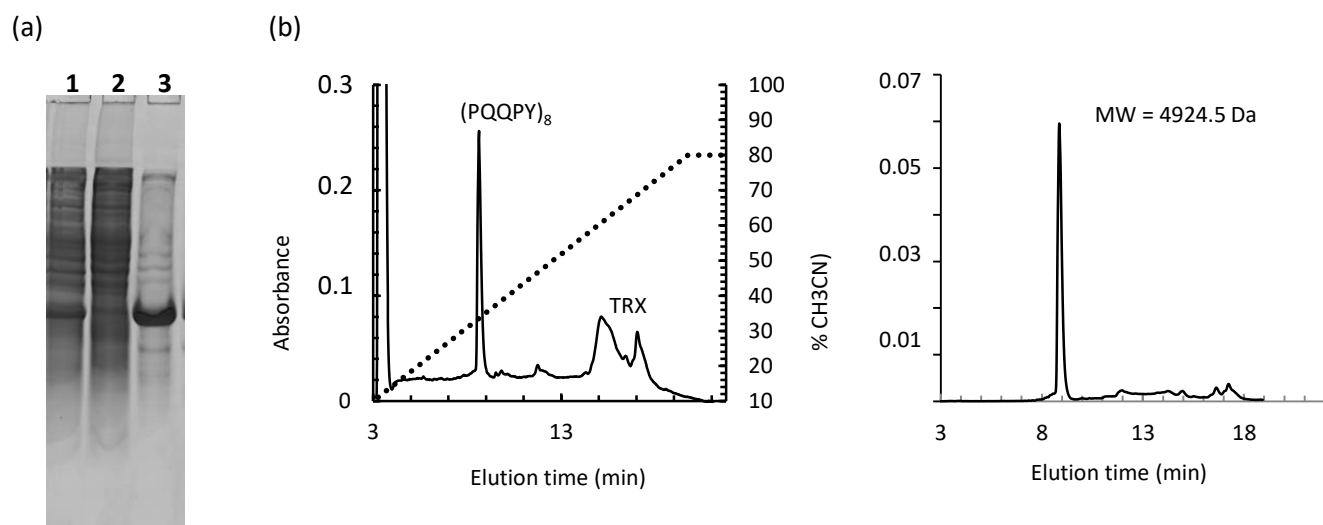

Supplement: S2 Fig — (a) Coomassie blue stained SDS–PAGE of (1) total proteins of the BLR strain carrying pETb (PQQPY)8 plasmid, (2) non-adsorbed proteins, (3) and eluted TRX-(PQQPY)8 polypeptide of the affinity chromatography. (b) HPLC of TRX-(PQQPY)8 cleavage reaction in 70% formic acid (5 mg.mL-1) (left) and the acid reaction products purified through a C18-T cartridge (right). (PDF) [file pone.0225293.s002.pdf]

**S3 Figure.**

**(a)**

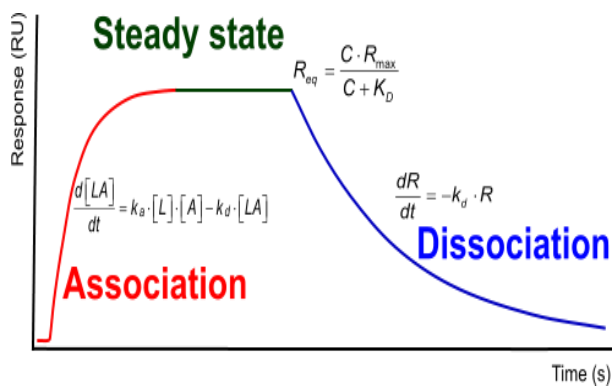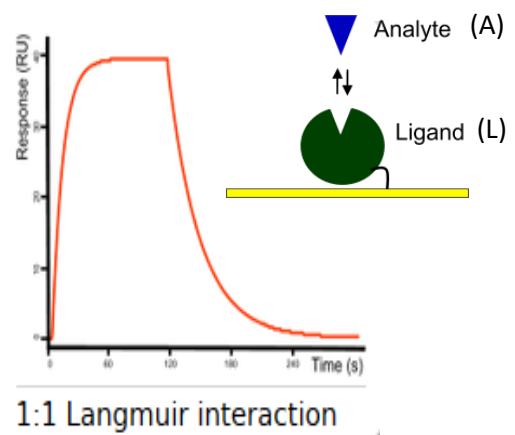

**(b)**

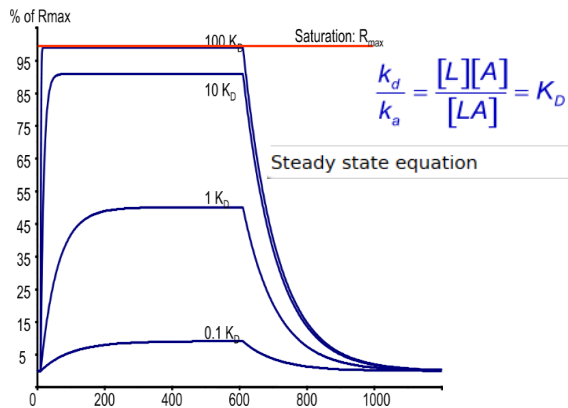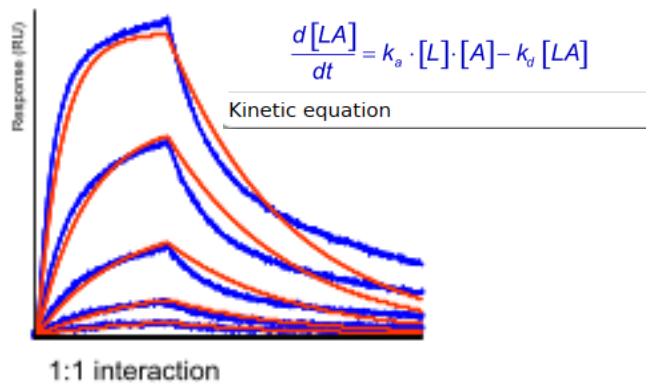

Supplement: S3 Fig — (a) Association: two or more molecules bind to each other. Steady state: the number of molecules that is binding is equal to the amount of bonds that is breaking. Dissociation: the breaking of the bounds between the molecules. (b) The kinetic parameters (ka: association rate; kd: dissociation rate; KD = kd/ka: equilibrium dissociation constant) were determined by globally fitting the experimental data using BIA-evaluation software 4.1 (GE Healthcare) with available binding models (1:1 Langmuir model). The binding constants were calculated by two methods: affinity analysis (steady state fit) and association/dissociation analysis (kinetic fit). (PDF) [file pone.0225293.s003.pdf]

S4 Figure.

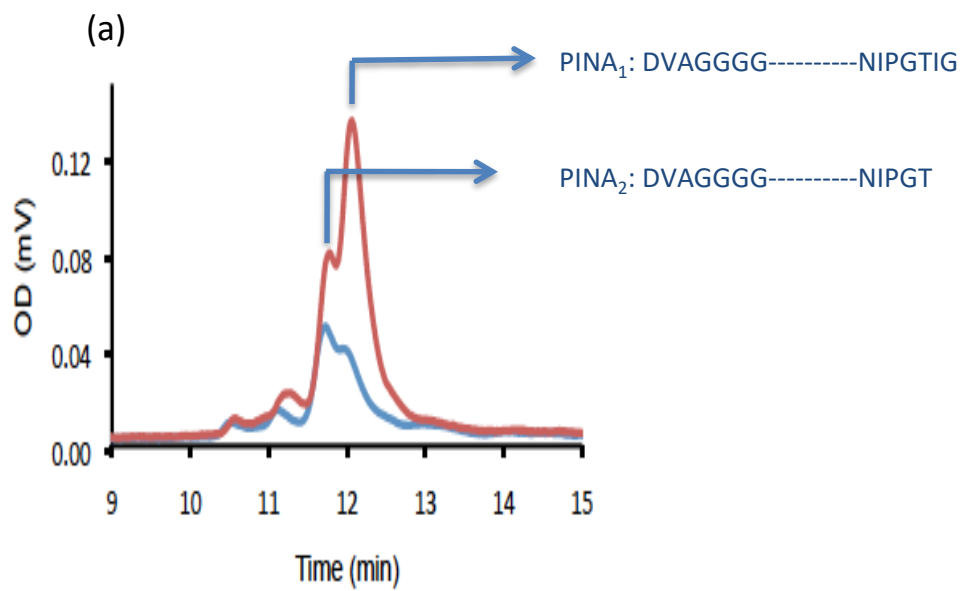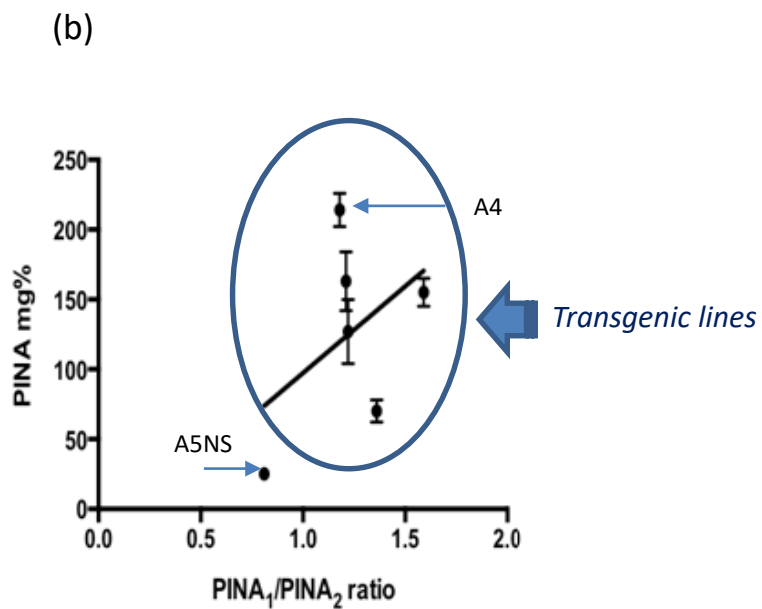

Supplement: S4 Fig — (a): RP-HPLC of PINA from transgenic line A4 overexpressing PINA (red line) and from null segregant A5NS (blue line).(b) ratio of the integrated peak surfaces corresponding to the two major post-translational processed PINA, PINA1 and PINA2. Mass spectrometry characterization of the different peaks was previously described [20]. (PDF) [file pone.0225293.s004.pdf]

S5 Figure.

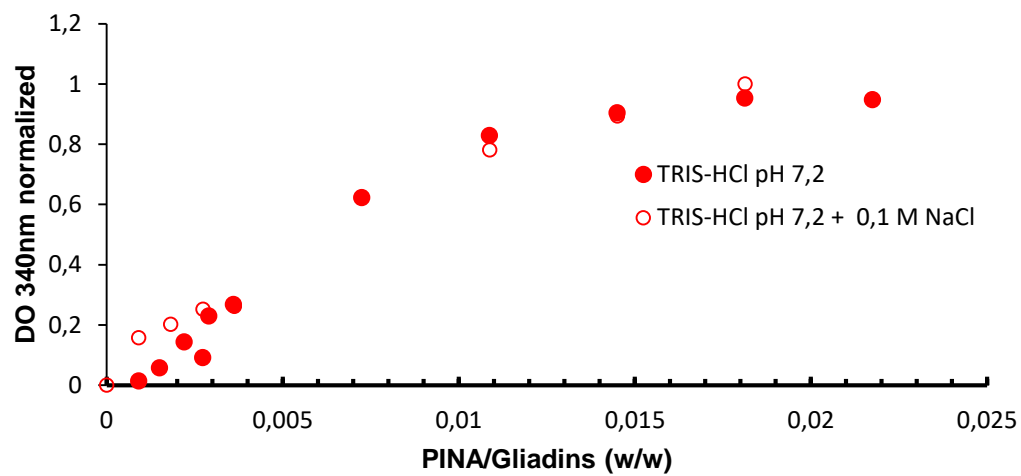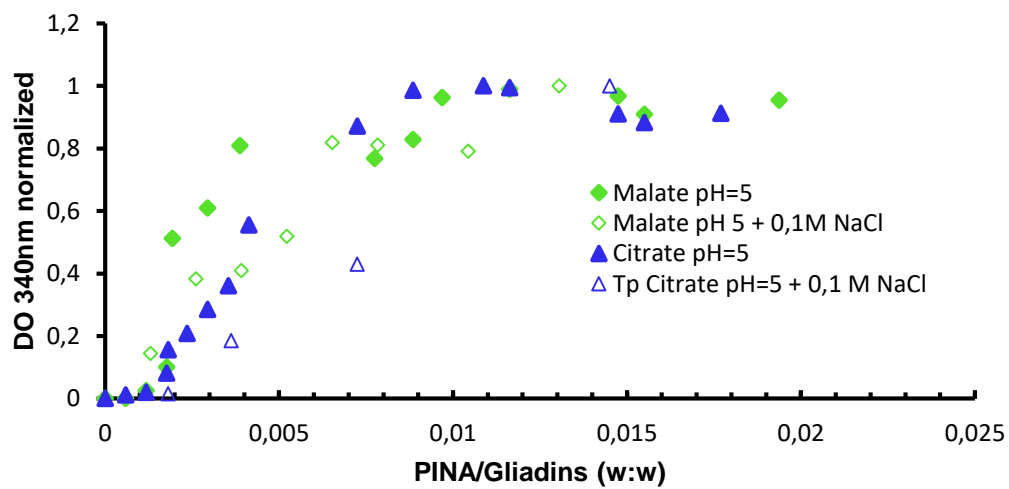

Supplement: S5 Fig — (PDF) [file pone.0225293.s005.pdf]

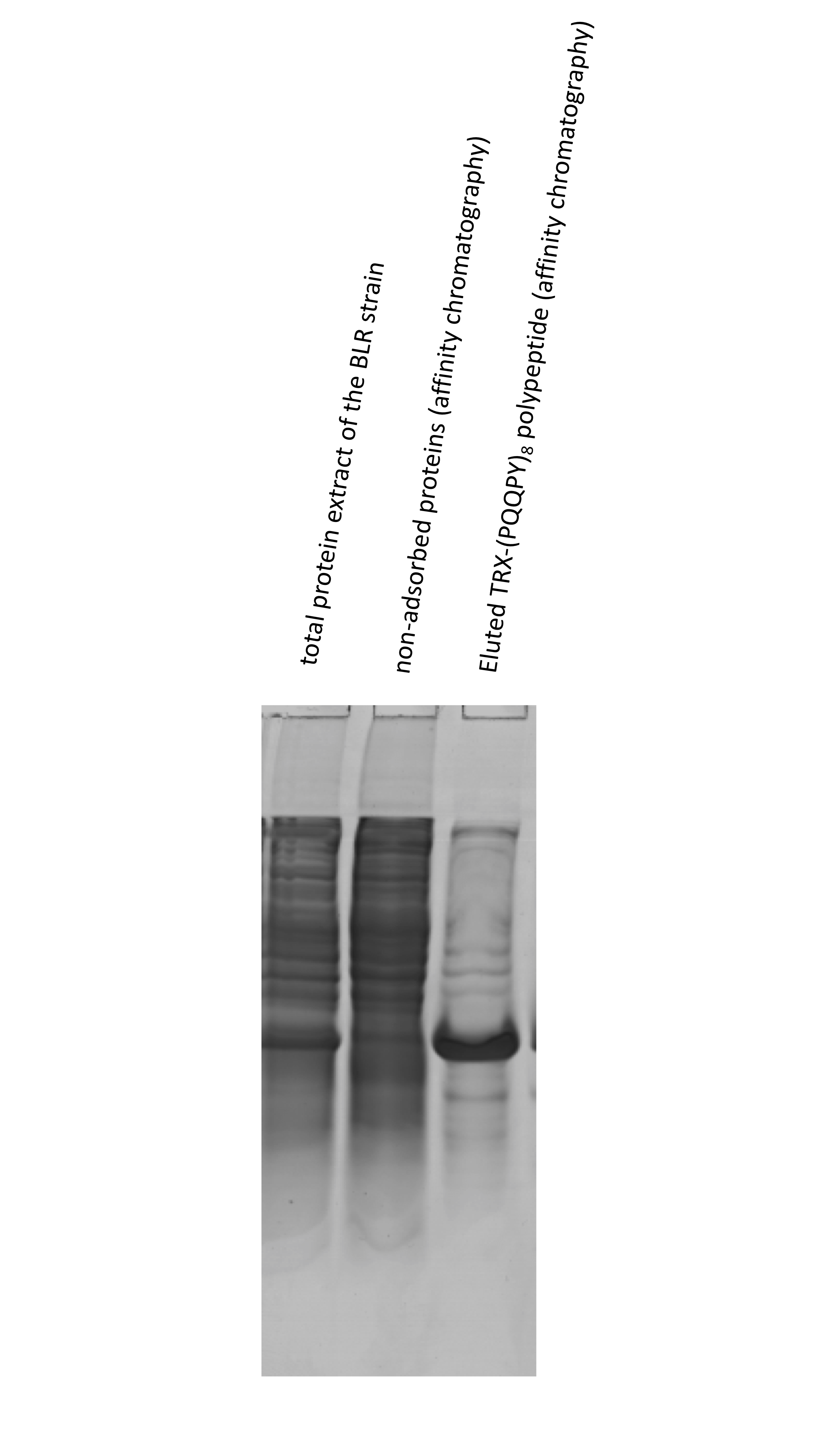

Supplement: S2 Raw image — (TIFF) [file pone.0225293.s007.tiff]
